# Cell Receptor and Cofactor Interactions of the Contact Activation System and Factor XI

**DOI:** 10.3389/fmed.2018.00066

**Published:** 2018-03-21

**Authors:** Monika Pathak, Bubacarr Gibril Kaira, Alexandre Slater, Jonas Emsley

**Affiliations:** ^1^Centre for Biomolecular Sciences, School of Pharmacy, University of Nottingham, Nottingham, United Kingdom

**Keywords:** contact activation system, factor XII, factor XI, plasma kallikrein, high molecular weight kininogen, endothelial cell, Platelet, Leukocyte

## Abstract

The contact activation system (CAS) or contact pathway is central to the crosstalk between coagulation and inflammation and contributes to diverse disorders affecting the cardiovascular system. CAS initiation contributes to thrombosis but is not required for hemostasis and can trigger plasma coagulation *via* the intrinsic pathway [through factor XI (FXI)] and inflammation *via* bradykinin release. Activation of factor XII (FXII) is the principal starting point for the cascade of proteolytic cleavages involving FXI, prekallikrein (PK), and cofactor high molecular weight kininogen (HK) but the precise location and cell receptor interactions controlling these reactions remains unclear. FXII, PK, FXI, and HK utilize key protein domains to mediate binding interactions to cognate cell receptors and diverse ligands, which regulates protease activation. The assembly of contact factors has been demonstrated on the cell membranes of a variety of cell types and microorganisms. The cooperation between the contact factors and endothelial cells, platelets, and leukocytes contributes to pathways driving thrombosis yet the basis of these interactions and the relationship with activation of the contact factors remains undefined. This review focuses on cell receptor interactions of contact proteins and FXI to develop a cell-based model for the regulation of contact activation.

## Introduction

**Figure 1:**
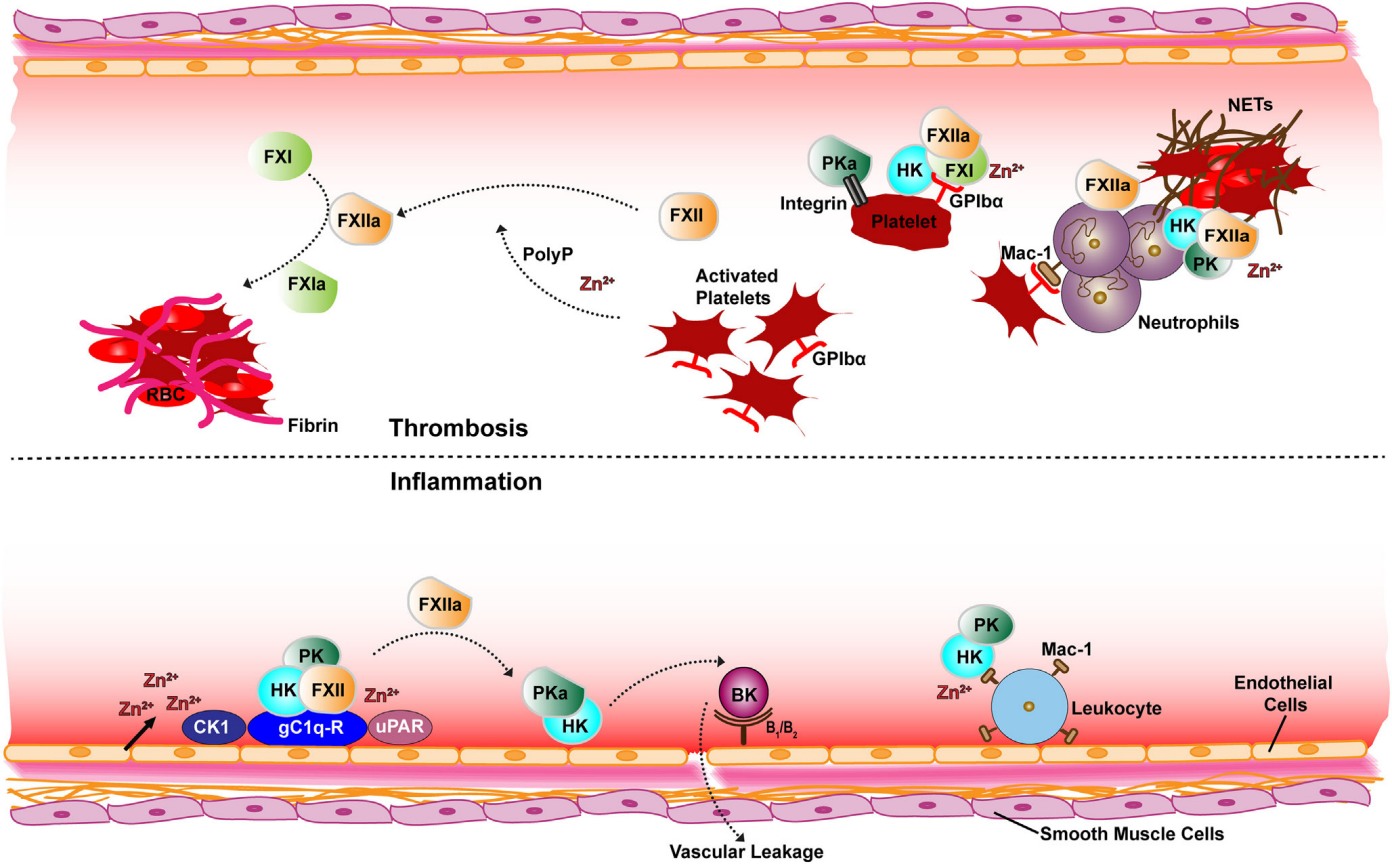
Schematic overview of receptor and cofactor interactions of the contact factors for thrombotic (top) and inflammatory (bottom) pathways. Assembly of the contact system *via* gC1q-R (with elevated Zn^2+^) generates PKa, FXIIa, and BK is produced on the surface of endothelial cells (bottom). Also shown is the FXII and HK interactions with the uPAR receptor and CK1. FXIIa activates plasma coagulation cascades *via* FXI on the surface of platelets (top). The platelet GP1b-IX receptor GP1bα chain interaction with FXI–HK and PKa binding to the activated platelet integrin αIIbβ3 is also depicted. Neutrophils are also depicted releasing NETs known to associate with contact proteins. Abbreviations: FXII, factor XII; FXIIa, activated FXII; PK, prekallikrein; PKa, activated PK; FXI, factor XI; FXIa, activated FXI; HK, high molecular-weight kininogen; BK, bradykinin; uPAR, urokinase receptor; GPIbα, platelet glycoprotein Ib; CK1, cytokeratin 1; gC1q-R, receptor for complement protein C1q; Mac-1, macrophage-1 antigen receptor; PolyP, polyphosphate; RBC, red blood cells; NETs, neutrophil extracellular traps.

The contact activation system (CAS) includes serine proteases factor XII (FXII), plasma prekallikrein (PK), coagulation factor XI (FXI), and high molecular weight kininogen (HK) which is the non-enzymatic cofactor of FXI and PK (1, 2). The CAS is thought to be central to crosstalk between coagulation and inflammation and the underlying cause for various disorders affecting the cardiovascular system (1, 3). Two branches of the CAS have been identified as (i) the inflammatory branch activates contact factors FXII and PK on the surface of endothelial cells resulting in release of the peptide bradykinin (BK) and (ii) plasma coagulation branch activates FXII and FXI on the surface of platelets (Figure 1) (4–6). Contact of FXII with diverse negatively charged activators leads to a change in the conformation of FXII that subsequently generates activated FXII (FXIIa) in trivial amounts (7–10). FXIIa then activates PK to form active kallikrein (PKa) (11). Reciprocal activation of FXII by PKa and PK by FXIIa occurs and subsequently PKa proteolytically liberates BK *via* cleavage of its precursor HK. Binding of BK to its receptor on endothelial cells results in activation of several pro-inflammatory signaling pathways leading to vasodilation, pain, and neutrophil chemotaxis (12, 13). CAS factors also participate in fibrinolytic and angiogenic pathways (14, 15).

Contact activation is best known as the coagulation mechanism that is activated by artificial surfaces and is the basis of the widely used aPTT hemostatic assay (2). Negatively charged polymers including nucleic acids (DNA and RNA) and polyphosphate (PolyP) are activators of the contact pathway *via* FXII auto-activation (16–19). Platelet-derived PolyP is mainly secreted as short chain polymers following platelet activation and has been linked to FXII activation and thrombus formation (20). Activated platelets also retain PolyP on their cell surface (membrane-associated) assembled into nanoparticles that can potently activate FXII (20). Purified DNA and RNA have been shown to bind and activate contact factors and enhance thrombin generation and clot formation in plasma based studies (21, 22). Genetic knockout studies in murine models of cardiovascular disease and genetic linkage studies in humans have implicated the contact factors in contributing to diverse cardiovascular disease processes, including thrombosis (23–29), hypertension (30), atherosclerosis (31, 32), and stroke (33, 34).

CAS factors are not considered vital for normal hemostasis *in vivo* as evidenced by patients with FXII deficiency exhibiting no bleeding tendencies (35, 36). However, some cases of FXI-deficiency in humans manifest with a strong bleeding phenotype (37). The first clinical trial revealed prevention of venous thrombosis by targeting FXI without compromising normal hemostasis (38, 39). In humans identification of a gain-of-function mutation in the *F12* gene (encodes FXII) was shown to be linked to aggressive attacks of tissue swelling in hereditary angioedema, a rare life threatening inherited edema disorder in which excessive formation of BK leads to recurrent episodes of acute swelling and increased vascular permeability (40, 41). In this context the presence of a hyperactive FXII mutant does not seem to cause thrombosis in these patients which is consistent with previous observations that the FXII–PK–HK branch and BK production can operate separately from FXI activation and plasma coagulation (40). What leads to this mechanistic uncoupling of FXIIa driven thrombosis and inflammation is unknown raising the question as to how the proteases are regulated and highlights the importance of understanding the precise cell receptors utilized for CAS regulation.

The contact factors each have individual properties including recognizing foreign substances and interacting with different cell types (29, 32, 42, 43) and bacteria (44–46). CAS has been implicated in viral pathogenesis (47) and as a component of the innate immune response (48). The assembly of contact factors has been demonstrated on the membranes of a variety of cell types and in this review we summarize the known cell receptor and cofactor interactions of contact factors and FXI.

## Contact Factor Domain Structure

It is well established that the vitamin K-dependant coagulation factors, such as pro-thrombin, factor X, and factor IX (FIX), utilize Gla domains and calcium ions (Ca^2+^) to recognize the phosphatidylserine exposed on activated cell membranes and these interactions control efficient proteolytic cleavage and activation of these factors on a specific surface (49, 50). Contact factors FXII, PK, and FXI do not have Gla domains but instead utilize key protein modules of apple and fibronectin-like domains to mediate binding interactions to cognate cell receptors and diverse ligands, which appropriately regulate their substrate recognition and enzyme activation. Another key difference is that zinc (Zn^2+^) ions are implicated as critical to the function of the contact factors as opposed to Ca^2+^ ions, which are required for Gla domain structure and binding to cell membranes.

### Factor XII

Factor XII is a 80 kDa glycosylated protein consisting of a single polypeptide chain and circulates in plasma as a zymogen with a concentration of 40 μg/ml (375 nM). Upon contact with anionic surfaces, in the presence of Zn^2+^ ions, FXII undergoes a conformational rearrangement leading to auto-activation or cleavage in *trans* by kallikrein to generate FXIIa. FXIIa consists of two chains; an N-terminal 52-kDa heavy chain and a C-terminal 28-kDa serine protease domain (51), linked together by a disulfide bond. The domain structure of the FXII heavy chain is composed of an N-terminal fibronectin type-II domain (FnII), an epidermal growth factor like domain (EGFI), a fibronectin type-I domain (FnI), a second EGF-like domain (EGFII), a kringle domain, and a distinctive proline-rich domain (52). The FXII heavy chain mediates binding to Zn^2+^ ions and negatively charged surfaces (53–55). FXII has been shown to bind to the urokinase receptor (uPAR) and platelet glycoprotein Ib (GPIb-IX) receptor (56, 57).

### Prekallikrein

Prekallikrein is a glycoprotein of molecular weight 88 kDa consisting of a single polypeptide chain that circulates in plasma as a zymogen at a concentration of 50 μg/ml (490 nM), with an estimated 75% bound non covalently to HK (58). FXIIa or β-FXIIa (the isolated protease domain fragment) cleaves PK resulting in a two-chain enzyme kallikrein (PKa), consisting of a 52-kDa heavy chain and 33–36 kDa light chain corresponding to the serine protease domain and both chains are linked together by a disulfide bond. PK shares 58% amino acid sequence identity with FXI and both proteins have the characteristic feature of four apple domains (A1–A4) (59, 60). The HK binding region on PK is localized in the central portion of the A2 domain with possible binding sites in other apple domains (61). PK binds to endothelial cells, platelets, and granulocytes in a Zn^2+^-dependent interaction *via* the PK–HK complex (2).

### High Molecular Weight Kininogen

High molecular weight kininogen is a 120 kDa non-enzymatic glycoprotein with a plasma concentration of 80 μg/ml (670 nM). Granulocytes, platelets, and endothelial cells contain HK, but plasma HK is most likely synthesized in the liver. HK is made up of six domains (D1–D6) and cleavage of HK by kallikrein PKa results in HKa, a two-chain protein consisting of a heavy chain (D1–D3; 64 kDa) and a light chain (D5–D6; 56 kDa) releasing the short BK peptide (D4) (62). The D6 domain has binding sites for PK and FXI (63). HK binds to cell surfaces in a Zn^2+^-dependent manner (64, 65). Both D5 and D3 domain mediate cell receptor binding of HK to endothelial cells, platelets, and neutrophils (62, 64, 66, 67). HK is constitutively bound to cell surfaces and mediates not only recruitment of PK and FXI to cell membranes but also functions to enhance interactions between different cell types (68, 69).

### Factor XI

Factor XI is a dimer of 80 kDa subunits that circulates in plasma at a concentration of 5 μg/ml (30 nM) tightly bound in a non-covalent complex with HK (70). Activation of FXI by thrombin or α-FXIIa yields FXIa that consists of a heavy chain of four apple domains (A1-A4) and a light chain of the catalytic serine protease domain covalently linked together *via* a disulfide bond. Apple domains A1 and A2 contain a binding site for thrombin and HK, respectively (50, 59). A3 has a FIX, heparin, and GPIb-IX binding site and A4 contains a cysteine residue that forms the disulfide bond needed for FXI dimer formation (42). FXI–HK complex binding to platelets has been reported to occur *via* the GPIb-IX receptor (71).

## Cell Interactions of Contact Factors

The interaction of contact factors with different cell types has been shown to cooperatively contribute to thrombotic pathways in animal models (29). Despite decades of research investigating *in vitro* characterization of CAS activation by polymers (15, 52), a detailed understanding of the receptors and cofactors that regulate CAS protease activation on cellular surfaces remains elusive. Figure 1 summarizes the key cell interactions of contact factors and below we consider each cell specific interaction in turn.

### Endothelial Cells

The binding of the contact factors to endothelial cells is important to localize the production of the vasoactive peptide BK to the correct surface where its receptors are located. HK is bound tightly to PK and FXI but there is evidence that PK and FXI can also bind endothelial cells in the absence of HK (72, 73). One of the major endothelial cell receptors that binds HK and FXII is gC1q-R (receptor for the complement protein C1q) (74–76), also known as p32 or hyaluronic acid binding protein 1. Most human cell types, including lymphocytes, endothelial cells, and dendritic cells express gC1q-R. gC1q-R is a multi-compartmental, multi-ligand binding cellular protein localized predominantly in the mitochondria as well as the plasma membrane, cytosol, Golgi, endoplasmic reticulum, and the nucleus (77–79). gC1q-R is present in the mitochondrial matrix where it binds and inhibits cellular splicing factors (80) and is also observed to inhibit splicing in HIV transcripts (81). In *Drosophila* embryos a fly ortholog of gC1q-R was identified as a histone chaperone that exchanges protamines for histones (82). Human gC1q-R binds with high affinity to all subclasses of histones (H1, H2A, H2B, H3.1, and H4) and blocks the pathophysiological activities of histones in a murine model for histone-induced shock (83). gC1q-R also binds host-defense peptides (84). In endothelial cells, inflammatory mediators and lipopolysaccharides (LPS) upregulate expression of gC1q-R in a time and concentration-dependent manner (85). gC1q-R was first discovered as binding the globular heads of the complement protein C1q (74) and is implicated in diverse biological pathways, including adipogenesis and insulin signaling (86), regulation of RNA splicing (80), the proliferation of tumor cells (87) and atherosclerosis (88).

The interaction of gC1q-R (89) with both PK–HK and FXII on the surface of endothelial cells promotes the production of PKa and FXIIa as gC1q-R is an endogenous activator of CAS (90). Biochemically, the properties of gC1q-R are consistent with this as it is a highly anionic multimeric protein capable of binding to both FXII and HK (78, 91) in a Zn^2+^-dependent manner (74, 76). Binding studies calculated a *K*_D_ of 0.7–0.8 nM for the interaction of HK with gC1q-R, and no difference in binding affinity was observed between HK and HKa (92). Previous studies suggest that FXII and HK compete with one another for binding to gC1q-R and for the same site on endothelial cells (43, 93, 94).

**Figure 2:**
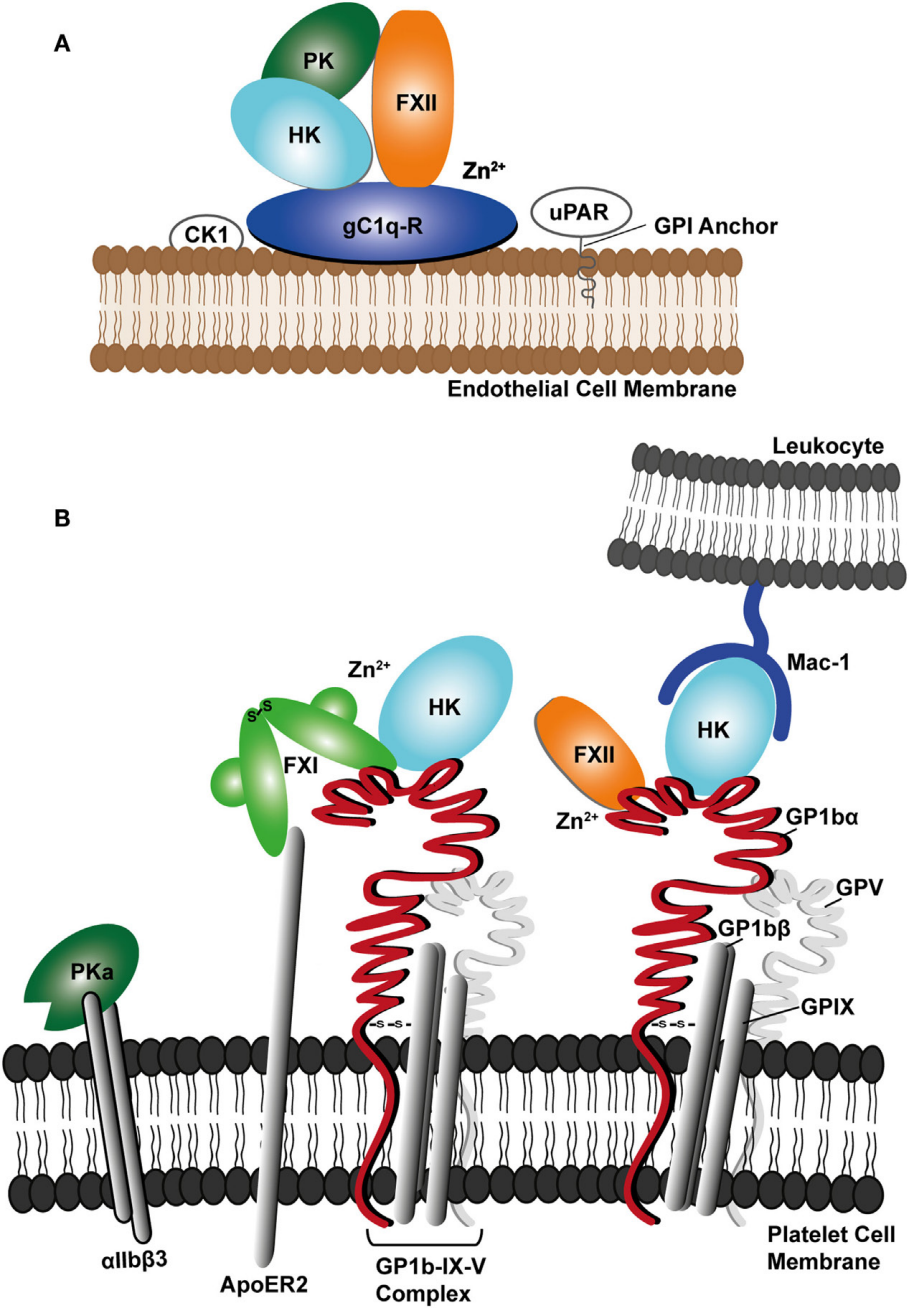
Schematic diagram showing **(A)** assembly of contact factors factor XII (FXII), prekallikrein (PK), and cofactor high molecular weight kininogen (HK) with the endothelial cell receptor complex involving the receptor for the complement protein C1q (gC1q-R), urokinase receptor (uPAR), and cytokeratin 1 (CK1). **(B)** Platelet receptor glycoprotein Ib complex (GPIb-IX-V) is shown forming a network of interactions with FXII, FXI, and HK and the Mac-1 receptor on leukocytes, where HK may bridge the two cell types. PKa is shown binding to the activated αIIbβ3 platelet integrin.

CAS can be regulated by gC1q-R as in the resting state gC1q-R is predominantly found in the cytoplasm and mitochondria and is only released to the cell surface upon activation of endothelial cells (95, 96). However, gC1q-R alone is not sufficient for full CAS activation as elevated Zn^2+^ ions are required for FXII recruitment to the complex. The origin of the Zn^2+^ has been shown to come from endothelial cells or activated platelets (43). Additional endothelial cell receptors for the contact factors are uPAR and cytokeratin 1 (CK1) (43, 69). These two proteins have been reported to form a multiprotein complex with gC1q-R on the surface of endothelial cells (Figure 2A), which is able to bind FXII (97). Additionally, the complex of uPAR with CK1 was shown to bind HK resulting in PK activation at the endothelial cell surface (98).

uPAR is a well characterized protease receptor and regulates the amount of active plasmin generated at the cell surface, through the interaction with urokinase (uPA) (99, 100), resulting in the degradation of fibrin fibers (101). Through this mechanism, uPAR has been linked with a number of biological processes, including cell migration, angiogenesis (102), tumor metastasis (103), and leukocyte migration (104). HK binding with uPAR inhibits endothelial cell migration and proliferation, and angiogenesis by disrupting the interaction of uPAR with uPA (69, 105, 106). While it is well established that HK/HKa binds to uPAR, Betapudi *et al*., (107) showed that the antiangiogenic effects of HK are mediated equally well in wild-type and uPAR-deficient mice, concluding that uPAR is not essential for inhibition of angiogenesis by HKa *in vivo* or for HKa-induced endothelial cell apoptosis *in vitro*. This study also failed to demonstrate an essential role for any of the previously known endothelial cell receptors for HK or HKa including the uPAR, gC1q-R, and CK1. Also, HKa inhibits angiogenesis *via* induction of apoptosis in proliferating endothelial cells and these effects were mediated mostly by HKa domain D5. A separate study showed an interaction between HKa domain D5 and endothelial tropomyosin underlies the antiangiogenic activity of HKa (108), indicating there is likely redundancy in the mechanisms whereby HK binds to cell surfaces.

A study using surface plasmon resonance has measured the binding of HK to these endothelial receptors revealing that HK binds with the greatest affinity to gC1q-R (0.8 nM), followed by CK1 (15 nM) and then uPAR (2.3 µM), each in a Zn^2+^-dependent manner (92). Both gC1q-R and CK1 showed no significant differences in binding affinity for HKa or HK, whereas uPAR bound 50-fold tighter to active HKa. It was, therefore, proposed that gC1q-R and CK1 are involved in the initial binding of HK to the cell surface where it is then cleaved by PKa. HKa is then able to selectively bind uPAR and mediate cell migration. As gC1q-R does not have a membrane anchor, additional interactions are likely critical to position the protein in the correct location on the endothelial cell surface to bind HK and FXII.

### Platelets

GPIb-IX is the receptor on platelets for FXII (57), FXI (109) and HK (110, 111). GP1bα is a subunit of the GPIb–IX complex, which plays a prominent role in the initial steps of platelet adhesion (112). The GPIbα–FXI interaction has been demonstrated biochemically to be mediated by the FXI A3 domain in a Zn^2+^-dependent fashion (*K*_d_ ~ 52 nM). The Interaction is localized to the GPIbα N-terminal leucine-rich repeats at a site distinct to the GPIbα anionic region, and FXI binding was shown to compete with VWFA1 but not thrombin binding (71, 109, 113). Recent studies on the FXI-GPIbα receptor interactions describe a vascular coagulation and inflammatory circuit that overlaps with arterial hypertension pathways (30). The apolipoprotein E receptor 2 (ApoER2, LRP8) has also been identified as a platelet receptor for FXI (114). ApoER2 is a member of the low-density lipoprotein family of receptors and initiates platelet cell signaling through the disabled-1 adaptor protein (115). Due to the dimeric nature of FXI, it may simultaneously bind both GPIbα and ApoER2.

GPIbα is also the primary receptor on platelets for thrombin and thus plays a well characterized role in platelet activation (116). HK binding to platelets is mediated predominantly through GPIbα (117) and this binding has been shown to compete with the thrombin-binding site, which is localized to the GPIbα anionic region (118). HK has been shown to interact with GPIbα through the D3 domain and the D5 domain although the precise determinants of the interaction are not clear (117). A monoclonal antibody binding to the GPIX subunit also inhibited HK binding to platelets, indicating there may be a more extensive interaction beyond the GPIbα chain (117).

FXII binds to GPIbα and in a similar manner to HK and FXII has also been shown to inhibit the binding of thrombin (57). As both FXII and HK have domains with concentrated regions of positive charge, it is possible both recognize the GPIbα N-terminal domain anionic region in a similar fashion to the way thrombin utilizes its positively charged exosites to bind GPIbα (57, 117, 118). The platelet membrane is well known as a pro-coagulant surface and a component of this response is the feedback loop of thrombin to cleave and activate FXI (119). PK binds platelets and recently the activated enzyme PKa has been described binding to platelet integrin αIIbβ3 through its KGD and KGE motifs (Figure 2) (120). PKa (but not the zymogen) enhances ADP induced platelet activation by PAR-1 hydrolysis (120).

### Leukocytes

HK, PK, FXII, and FXI have been shown to bind to the surface of neutrophils (121). Thus, release of BK on the neutrophil surface could enhance the passage of neutrophils out of the vasculature to mediate inflammatory responses (121). HK inhibits calpain (122), is involved in neutrophil aggregation (123), chemotaxis (124), and the release of elastase through degranulation (124, 125).

The major leukocyte cell receptor for HK is the activated integrin macrophage-1 antigen receptor (Mac-1) (66). HK binds to Mac-1 *via* the D3 and D5 domains (66, 126). Mac-1 is a multifunctional receptor expressed primarily on monocytes, macrophages, neutrophils, and natural killer cells. A variety of different ligands bind to this receptor including fibrinogen (66), intercellular adhesion molecule-1 (ICAM-1) (127) as well as HK (66). Mac-1 also mediates a variety of cell to cell interactions, including the neutrophil-platelet association involving interaction with GPIbα (128). HK mediates the adhesion of neutrophils to sites of fibrin formation and endothelial cells by inhibiting the interaction of Mac-1 with fibrinogen and ICAM-1 (129). Surfaces preadsorbed with HK are anti-adhesive to neutrophil binding (130).

Platelets and leukocytes interact and coordinate innate immune responses (131). The relationship between these two cell types and the CAS has been described as being involved in a pathway termed immuno-thrombosis that can contribute to thrombus formation in animal models of disease (132). Thus, it is of interest that HK binds both cell types and has been described as a molecular bridge between GPIbα and Mac-1 enhancing the interaction between cell types through these receptors (Figure 2B) (68). Mac-1 has also been linked to neutrophil extracellular traps (NETs) formation (133, 134). This promotes microbe entrapment by fibrin clots thus facilitating microbial clearance through the engagement of phagocytic cells and leukocytes by stimulating inflammation. Thus, HK may be a key coordinating cofactor for drawing together the CAS plasma proteins and different cell types in an innate immune pathway that overlaps with thrombosis (29).

### Contact Factor Interactions with Bacteria and Viruses

Negatively charged LPS or surface associated negatively charged teichoic acids (*S. aureus*) (135, 136) and long chain PolyP (137) from various bacteria can induce CAS activation and BK release (138). HK binding to LPS from *K. pneumoniae, P. aeruginosa, S. Minnesota*, and *E. coli* strains converts single-chain HK to two-chain HKa and releases BK (139). High levels of BK have been reported in animal models of sepsis (140).

Contact factors bind to the surface of Gram-negative bacteria as well as Gram-positive bacteria and FXII and HK dependent contact activation on fibrous structures, including curli and fimbriae in *E. coli* and *S. typhimurium*, respectively, activates CAS which is not found in mutant strains lacking either curli or fimbriae (141, 142). The streptococcal M1 protein together with human fibrinogen, initiates polymorphonuclear neutrophils to form NETs, providing a surface for binding and activation of the contact system (143).

FXII mediated contact activation (144) and coagulation during viral infections has been reported (48). Upon hantavirus infection increased FXII binding and auto-activation is observed on the surface of infected endothelial cells (47). In Herpes Simplex Virus 1 an anionic phospholipid was identified as being responsible for activating FXII leading to enhanced coagulation through CAS activation (145).

## Conclusion

The assembly of contact factors on cell surfaces is mediated *via* a number of structurally unrelated cell receptors (uPAR, GPIbα, Mac-1) and cofactors (HK, gC1q-R, CK1). On the surface of endothelial cells, gC1q-R is primarily responsible for assembly and activation of the FXII/HK/PK. gC1q-R has structural features of a cofactor rather than a cell receptor as it lacks a means to independently anchor to the plasma membrane and it is also present as a soluble factor in plasma (146). It has been shown by several groups independently that gC1q-R binds both HK and FXII and that it is capable of activating FXII and PK only when the three proteins, FXII/HK/PK, and elevated Zn^2+^ ions are present (90). Thus, it is capable of acting in a similar way to polyanions such as PolyP (90). As both gC1q-R and HK have been characterized as being capable of forming interactions with cell receptors and cell surface proteins (97, 147), there may be several redundancies in the mechanism whereby the FXII/HK/PK/gC1q-R complex locates to the surface of endothelial cells.

A key question is the molecular mechanism and pathophysiological significance of the Zn^2+^ dependence for both CAS receptor binding and FXII enzyme activation and whether this cation is a global regulator of CAS. The plasma concentration of Zn^2+^ ions ranges between 10 and 20 µM (148), most of which is bound to serum albumin, resulting in free Zn^2+^ concentrations of approximately 0.5 µM (149). FXII binding to endothelial cells increases in the presence of Zn^2+^ and binding plateaued at Zn^2+^ concentrations of 50 µM (93). This Zn^2+^ concentration is 100-fold more than the free Zn^2+^ concentration found in the plasma, which supports Zn^2+^ concentration as a potential control mechanism for contact activation. Platelets are known to release Zn^2+^ upon activation (43, 150) but the cellular process that controls this remains unknown. Different cell types cooperate in pathways leading to thrombosis (29, 30, 151) and thus studying each cell type in isolation may have limited utility when trying to understand contact factor cell localization and contact activation *in vivo*.

On the surface of platelets the assembly of the FXII/HK/FXI complex may occur *via* GPIbα, which can also act as the receptor for thrombin, thus coordinating FXI activation *via* both coagulation pathways (intrinsic and extrinsic). Activation of platelets and resulting secretion of PolyP plays a role both as a cofactor for thrombin cleavage of FXI (119) and FXII activation (17, 20, 152–155).

uPAR acts as a receptor for FXII on the surface of neutrophils (156) and dendritic cells (157), mediating processes that may be independent of contact activation. Contact factors are considered to be novel drug targets for thrombotic (36) and inflammatory diseases and targeting cell surface and receptor/cofactor binding domains has the potential of introducing more selectivity over targeting the contact factor enzyme active sites. Elucidating the structure of cognate cell receptors and cofactors regulating CAS activation in inflammation and FXI activation in the plasma coagulation pathway will provide a scaffold to develop novel antagonists and therapies for diverse vascular diseases.

## Author Contributions

All authors listed, have made substantial, direct and intellectual contribution to the work, and approved it for publication.

## Conflict of Interest Statement

The authors declare that the research was conducted in the absence of any commercial or financial relationships that could be construed as a potential conflict of interest.

## Abbreviations

CAS, contact activation system; PK, prekallikrein; HK, high molecular weight kininogen; FXII, factor XII; BK, bradykinin; uPAR, urokinase receptor; GPIb-IX, platelet glycoprotein Ib.
